# The chicken miR-150 targets the avian orthologue of the functional zebrafish MYB 3'UTR target site

**DOI:** 10.1186/1471-2199-11-67

**Published:** 2010-09-02

**Authors:** Audrey Guillon-Munos, Ginette Dambrine, Nicolas Richerioux, Damien Coupeau, Benoît Muylkens, Denis Rasschaert

**Affiliations:** 1 Université François Rabelais, Equipe Transcription, Lymphome Viro-induit, UFR Sciences et Techniques, Parc de Grandmont, F-37200 Tours, France; 2 INSERM, U618 Protéases et Vectorisation Pulmonaire, UFR de Médecine, F-37000 Tours, France; 3 INRA, Département de Santé Animale, F-37380 Nouzilly, France; 4 INRA, Laboratoire de Virologie Moléculaire, UR-IASP 213, F-37380 Nouzilly, France; 5 Université de Namur, Département Vétérinaire, Faculté des Sciences, FUNDP, 5000 Namur, Belgique

## Abstract

**Background:**

The *c-myb *proto-oncogene is the founding member of a family of transcription factors involved principally in haematopoiesis, in diverse organisms, from zebrafish to mammals. Its deregulation has been implicated in human leukaemogenesis and other cancers. The expression of *c-myb *is tightly regulated by post-transcriptional mechanisms involving microRNAs. MicroRNAs are small, highly conserved non-coding RNAs that inhibit translation and decrease mRNA stability by binding to regulatory motifs mostly located in the 3'UTR of target mRNAs conserved throughout evolution. MYB is an evolutionarily conserved miR-150 target experimentally validated in mice, humans and zebrafish. However, the functional miR-150 sites of humans and mice are orthologous, whereas that of zebrafish is different.

**Results:**

We identified the avian mature miRNA-150-5P, *Gallus gallus *gga-miR-150 from chicken leukocyte small-RNA libraries and showed that, as expected, the gga-miR-150 sequence was highly conserved, including the seed region sequence present in the other miR-150 sequences listed in miRBase. Reporter assays showed that gga-miR-150 acted on the avian MYB 3'UTR and identified the avian MYB target site involved in gga-miR-150 binding. A comparative *in silico *analysis of the miR-150 target sites of MYB 3'UTRs from different species led to the identification of a single set of putative target sites in amphibians and zebrafish, whereas two sets of putative target sites were identified in chicken and mammals. However, only the target site present in the chicken MYB 3'UTR that was identical to that in zebrafish was functional, despite the additional presence of mammalian target sites in chicken. This specific miR-150 site usage was not cell-type specific and persisted when the chicken *c-myb *3'UTR was used in the cell system to identify mammalian target sites, showing that this miR-150 target site usage was intrinsic to the chicken c-*myb *3'UTR.

**Conclusion:**

Our study of the avian MYB/gga-miR-150 interaction shows a conservation of miR-150 target site functionality between chicken and zebrafish that does not extend to mammals.

## Background

*c-myb *was originally identified as the chicken cell homologue of the *v-myb *oncogenes found in two strains of avian leukosis virus [1,2]. These avian *v-myb *oncogenes induce myeloid and erythroid forms of leukaemia in chickens and the activation of the *c-myb *promoter by the insertion of avian and murine retroviruses has also been implicated in diverse forms of leukaemia [3,4]. A role for MYB in human leukaemogenesis was initially suspected following the demonstration of MYB overproduction in cells from patients with leukaemia. This role has recently been confirmed by the detection of duplications and translocations affecting the *c-myb *locus, particularly in acute and chronic myeloid leukaemia and in acute T-cell lymphoblastic leukaemia [5,6]. MYB deregulation is also associated with colorectal cancers [7,8], carcinomas [9] and breast cancers expressing oestrogen receptor-alpha [10], in which MYB has been implicated in prolactin-induced signalling pathways [11].

In normal cells, MYB has been shown to be essential for haematopoietic lineage specification, T- and B-lymphocyte differentiation, colonic mucosal crypt regeneration and brain neurogenesis, on the basis of the abnormal phenotypes observed in mouse *myb *mutants [12,13]. In zebrafish, MYB has also been shown to be essential for haematopoiesis [14,15] and the silencing of *c-myb *in zebrafish embryos also leads to abnormal phenotypes, with effects on eye tissue formation in particular [16].

*c-myb *is the founding member of a family of genes encoding transcription factors with a DNA-binding domain consisting of three regions: R1, R2 and R3 [12]. Vertebrate genomes contain two other closely related genes from this family [17]: *MYBL1 *(also known as *A-myb*), which is expressed in a restricted panel of tissues, and *Mybl2 *(also known as *B-myb*), which is ubiquitously expressed. The products of these genes regulate the expression of genes involved in the control of cell proliferation and differentiation [18-20]. In plants, the R2R3 MYBs constitute a large family of transcription factors involved in the regulation of plant-specific developmental and physiological processes [21].

MYB generally acts as a transcriptional activator, binding to the MYB binding site on DNA [22] and recruiting the CBP/p300 coactivator proteins [23,24], thereby increasing the level of transcription of MYB targets. The oncogenic properties of MYB result from the overexpression or inappropiate activation of *c-myb *[13].

The level of *c-myb *expression is critical and is regulated by a number of mechanisms. At the transcriptional level, the *c-myb *promoter may be transactivated by a large number of proteins, including MYB itself, and attenuation sequences identified in the first intron of *myb *also regulate Myb elongation [13,25]. No particular pattern of cell specificity has been clearly demonstrated for transcriptional regulation processes, whereas tight control over the levels of mature MYB mRNA is restricted to tissue compartments with a high turnover. MYB mRNA and MYB protein have a very short half-life [13]. They are present in large amounts in haematopoietic progenitor cells but are absent from terminally differentiated cells, suggesting that rapid changes occur [26]. An absolute requirement for the fine-tuning of *c-myb *expression was recently highlighted by the demonstration of a compromising effect of decreases in *c-myb *gene activity on murine haematopoietic stem cells, leading to a myeloproliferative disorder involving stem cells with novel characteristics [27].

The post-transcriptional mechanisms regulating MYB levels involve microRNAs (miRNAs). miRNAs constitute a class of highly conserved small (21-24 nucleotides) non-coding RNAs found in plants and animals [28]. In animals, mature miRNAs generated by processing from the stem loop pre-miRNA are incorporated into the silencing complex (for a review see [28]), which mediates post-transcriptional repression by binding to mRNA molecules, causing a decrease in the rate of translation or stability of the target mRNA [28-32]. Metazoan miRNA target recognition is based on Watson-Crick pairing of the 5' region of the miRNA, mostly via nucleotides 2 to 8 -- known as the miRNA seed -- to sites generally located within the 3'-untranslated region (UTR) of the target mRNA. Several hundreds of miRNAs with highly conserved sequences have been identified in diverse vertebrate species, from fish to humans [28]. Moreover, the lengths and sequences of the 3' UTRs from mammals and birds are sufficiently similar for alignments to be generated [33]. Prediction algorithms (reviewed in [29]) based on base-pairing between the miRNA seed and mRNA sequences have identified potential miRNA target sites in thousands of human genes [34,35]. A comparative analysis of several mammalian genomes suggested that miRNA target sites were conserved regulatory motifs in mammals [33]. A pioneering comparative analysis of mammalian and fish miRNA targets yielded 240 orthologous miRNA target genes conserved between these two groups [34].

It has been predicted that miRNAs may regulate the production of proteins from as many as 10% to 30% of the genes present in the human genome [35]. There is evidence to suggest that miRNA function is critical for normal cellular development and homeostasis. The production of miR-150 in mature B and T cells has been shown to block early B-cell development, and its ectopic production in haematopoietic stem cells and progenitor cells has been shown to result in significantly lower than normal numbers of mature B cells [36]. Moreover, miR-150 controls B-cell differentiation by targeting murine MYB in a dose-dependent manner [37]. By targeting MYB, miR-150 also drives the differentiation of murine megakaryocyte-erythrocyte progenitors into megakaryocytes, indicating a key role for miR-150 in controlling lineage commitment [38]. Thrombopoietin also induces megakaryopoiesis by downregulating MYB expression through the effects of miR-150 [39]. Overall, in haematopoietic lineages and the B-cell subsets of tonsil tissues, miR-150 and MYB display opposite patterns of expression [40]. Opposite patterns of miR-150 and MYB expression are also observed in immortalised cell lines, in which no miR-150 is detected [41].

Like most vertebrate mRNAs [35], MYB mRNAs are conserved targets of microRNAs. Functional miR-150 target sites have been validated in human, murine and zebrafish MYB 3'UTRs [16,37] and orthologous functional target sites of miR-150 have been identified in mammals, but are not conserved in zebrafish.

We sought to increase knowledge about miR-150/MYB interactions during the course of evolution, by investigating the function of avian miR-150. Several attempts at extensive or deep sequencing [42-47] have generated a list of more than a hundred chicken miRNAs in miRbase, but only a few chicken targets have been validated [48-50]. The chicken, an amniote that has evolved separately from mammals for about 310 million years, has provided unique data on vertebrate evolution [51]. The evolutionary distance between chicken and mammals may eventually facilitate studies of the target site adaptation of mammalian miRNAs. We used a reporter assay to identify the functional targets of miR-150 in the avian MYB 3'UTR and performed a comparative *in silico *analysis of predicted and experimentally validated functional target sequences in the vertebrate MYB 3'UTR (frog, fishes, chicken, mammals). Our findings show that the number of putative miR-150 target sites has increased during evolution, with chicken and mammals displaying an additional set of target sites, but with only one of the sites in chicken being functional, that identical to the functional site in zebrafish. The avian miR-150 bound to the same target sites in chicken cells and in the human cell line used to identify mammalian miR-150 target sites, demonstrating the specificity of this pattern of binding to the avian MYB 3'UTR.

## Results and Discussion

### Cloning of mature avian gga-miR-150

**Figure 1:**
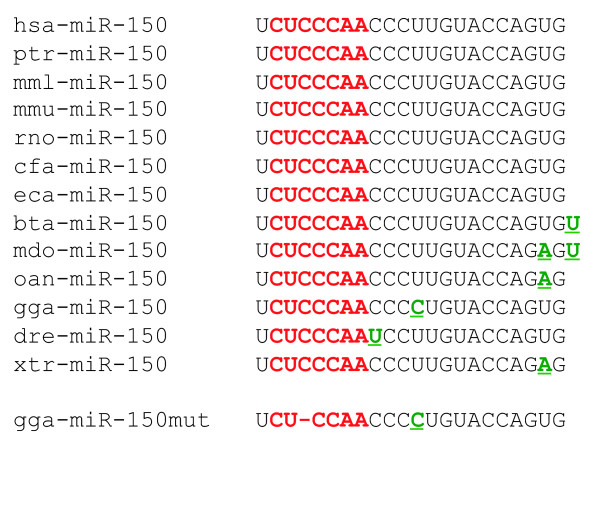
**Comparison of the sequence of avian gga-miR-150 with all miR-150 sequences from the miRBase**. The human sequence was taken as the reference sequence. The seed sequence is shown in red and changes are shown in green, underlined. Sequences were predicted on the basis of sequence similarity or experimentally validated. Hsa, *Homo sapiens*; ptr, *Pan troglodytes*; mml, *Macaca mulatta*; mmu, *Mus musculus*; rno, *Rattus norvegicus*; cfa, *Canis familiaris*; eca, *equus caballus*; bta, *Bos taurus*; mdo, *monodelphis domestica*; oan, *Ornithorhynchus anatinus*; gga, *Gallus gallus*; dre, *Danio rerio*; xtr, *Xenopus tropicalis*. The sequence of the mutated gga-miR-150 (gga-miR-150mut) that we constructed is shown.

We cloned avian mature miRNA-150-5P (*Gallus gallus*, gga-miR-150) from small-RNA libraries derived from spleen or peripheral blood leukocytes before and at advanced stages of Marek's disease virus (MDV)-induced lymphomagenesis in chickens (69 reads). By contrast, gga-miR-150 was not detected in libraries derived from chicken immortalised lymphoid cell lines [52,53]; [our unpublished data]. The expression pattern of the avian gga-miR-150 is similar to that of the human hsa-miR-150, which is expressed in normal human haematopoietic cell lineages but not in immortalised cell lines [16,41]. The sequences of miRNAs seem to have been conserved during evolution, because the mature gga-miR-150 cloned from our libraries and the recently released sequence [54]http://www.ncbi.nlm.nih.gov/geo/query/acc.cgi?acc=GPL6541 contain the seed region (nt2 to nt8) sequence (CUCCCAA) identified in the other twelve miR-150 sequences listed in miRBase [55,56]http://microrna.sanger.ac.uk (Fig. 1). All mature miR-150 sequences begin with a uracil (U) residue and only four positions have been found to harbour changes (Fig. 1). The mature gga-miR-150 sequence differed from its human and murine counterparts by one residue, and from the zebrafish sequence by two residues (Fig. 1).

### Chicken gga-miR-150 targets avian MYB

**Figure 2:**
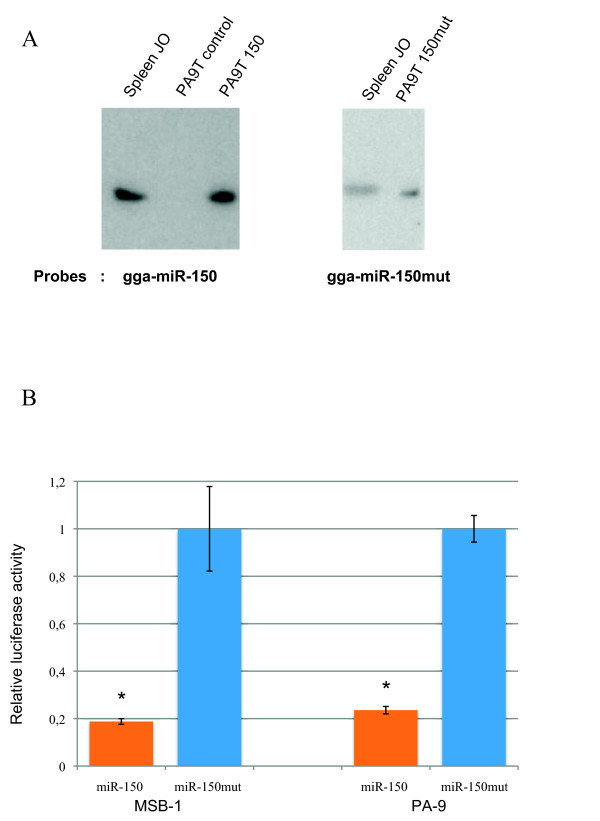
**gga-miR-150 ectopically produced in stable lymphoid chicken PA9 and MSB-1 cells represses the avian MYB reporter**. ***(A) ***gga-miR-150 and gga-miR-150mut detected by northern blotting. Positive control: spleen leukocytes from a 4-week-old chicken. Northern blots were performed with a ^32^P-5' end-labelled DNA oligonucleotide probe complementary to the miRNA. ***(B) ***The MYB reporter was repressed in PA9 or MSB-1 cells producing gga-miR-150. PA9 and MSB-1 cells were electroporated with 40 μg of avian MYB WT pRL-TK reporter and 500 ng of the normalising plasmid containing the firefly luciferase gene, pcDNAMLuc. Luciferase activity was determined 24 hours after transfection. *Renilla *luciferase activity was normalised with respect to firefly luciferase activity and luciferase activity in PA9 and MSB-1 cells producing gga-miR-150mut was set at 100%. The data from one experiment representative of the three carried out are shown, with all three assays generating essentially identical results (* significant repression, *P *< 0.05, Student's *t*-test comparisons with PA9T or MSB-1T150mut).

We investigated the targeting of avian MYB by gga-miR-150 in luciferase reporter assays on cell cultures. The sequence of the precursor pri-miRNA gga-miR-150 is not present in the released sequence of the *Gallus gallus *genome [57]http://www.ncbi.nlm.nih.gov/projects/genome/guide/chicken/ and we were unable to identify pri-miRNA gga-miR-150 on the basis of synteny with human and zebrafish. We therefore constructed a synthetic avian pre-miR-150, based on a comparison of various mammalian pre-miR-150 sequences and stem loop structures (Additional file 1). The synthetic avian pre-miR-150 amplicon obtained by PCR with appropriate primers (Additional file 2) was introduced into a pcDNA3 vector (pmiR-150). We used a miR-150 mutant plasmid (pmiR-150mut) as a control, with the deletion of one C residue within the miR seed sequence (Fig. 1), to prevent pairing between gga-miR-150 and putative targets in the avian MYB 3'UTR. We assessed the functionality of gga-miR-150, by establishing stable lymphoid chicken MDV cell lines ectopically producing gga-miR-150 or mutated gga-miR-150. The chicken MDV cell lines MSB-1 [58] and PA9 [59] were transfected with pmiR-150, pmiR-150mut or pcDNA vectors. MDV cells ectopically producing gga-miR-150 (MDVT-150) or mutated gga-miR-150 (MDVT-150mut) and control pcDNA-transfected cells (MDVT) were then isolated by selection on neomycin. Northern blots showed the ectopic production of a mature gga-miR-150 of the same size by MDVT-150 cells and spleen cells, whereas a slightly smaller mature mutated gga-miR-150 was detected in MDVT-150mut cells (Fig. 2A). These findings are consistent with the deletion of one nucleotide from the mature mutated gga-miR-150, confirming the successful transcription and maturation of synthetic avian pre-miR-150 from vectors with pol II promoters [60].

**Figure 3:**
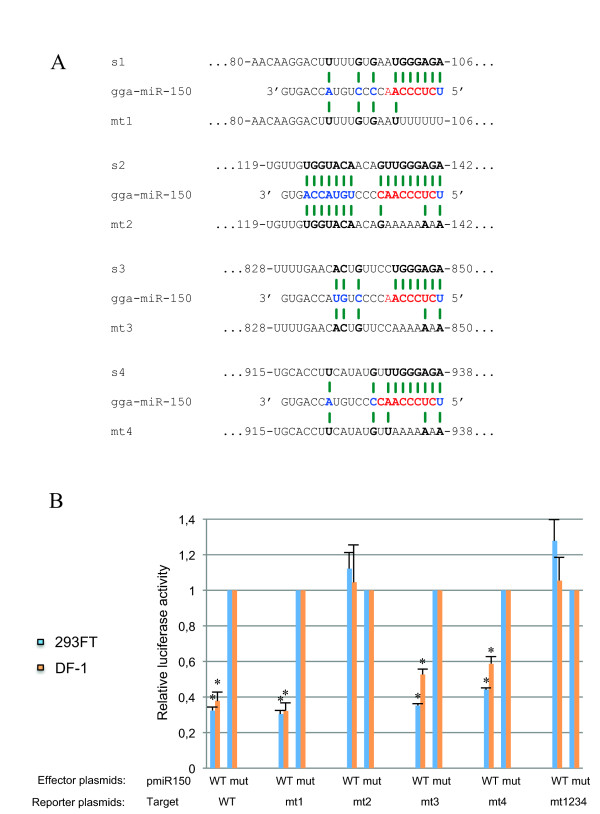
**The avian MYB 3'UTR contains a functional miR-150 target site**. ***(A) ***Predicted binding structures between gga-miR-150 and the four putative target sites (s1, s2, s3, s4) or their mutated counterparts (mt1, mt2, mt3, mt4) are shown. Numbers correspond to the position of the site in the avian MYB 3'UTR (nt1 = first nt downstream from the stop codon); nt in bold are involved in nt pairing. ***(B) ***gga-miR-150 repressed MYB reporters in chicken DF-1 cells or human 293FT cells through one specific target site. DF-1 or 293FT cells were cotransfected with 100 ng of reporter, 300 ng of effector and 1.25 ng of normalising pcDNAMLuc plasmid. Luciferase assays were performed as in Fig. 2 and luciferase activity following transfection with pmiR-150mut was set at 100%. Reporter plasmids: MYB WT reporter, MYB mt1, mt2, mt3, mt4, mt1234 reporters. Effector plasmids: pmiR-150, pmiR-150mut. The data presented are means ± SDs of three independent experiments (* significant repression, *P *< 0.05, Student's *t*-test comparisons with pmiR-150mut controls).

We investigated the targeting of avian MYB by gga-miR-150, by inserting the 3'UTR fragment of the chicken MYB cDNA into a pRL-TK vector downstream from the *Renilla *luciferase reporter gene. The various MDVT cells were cotransfected with the MYB WT pRL-TK vector and the internal control pcDNAMluc vector encoding firefly luciferase, by electroporation, and dual luciferase assays were performed. We normalised luciferase activity, taking the value for MDVT-150mut cells as 100%, as mutated gga-miR-150 did not repress activity in MDVT-150mut cells. The activity of the MYB WT reporter was specifically repressed by gga-miR-150 in PA9 and MSB-1T-150 cells (by up to about 80%) (Fig. 2B). We also transiently cotransfected avian DF1 fibroblasts with pmiR-150, pmiR-150mut or pcDNA plasmids and the MYB WT pRL-TK vector. Reporter assays in these cells confirmed that pmiR-150 efficiently reduced MYB WT reporter activity to about 60% (Fig. 3). Thus, in chickens, as in humans [16], the repression of MYB WT reporter activity by miR-150 does not seem to be cell type-specific and gga-miR-150, like its orthologues in humans, mice and zebrafish [16,37], specifically targets the 3'UTR of MYB.

### gga-miR-150 uses a target site orthologous to that of zebrafish

We identified four putative miR-150 target sites in the chicken MYB 3'UTR (Fig. 3A), all matching the criteria for miRNA target recognition and target site sequence context within the 3'UTR [35,61-63]. Sites s1 and s3, located 85-106 nt and 830-850 nt downstream from the stop codon, respectively, were 7-mer-A1 sites containing the seed sequence with an additional A residue in target position 1 (Fig. 3A). Such sites have been shown to be functional in various systems [63]. Sites s2 and s4, located 123-142 nt and 918-938 nt downstream from the stop codon, respectively, were 9-mer-A1 and 8-mer-A1 sites (Fig. 3A). These sites are probably at least as effective as the 8-mer sites shown to be functional in various systems, from worms to mammals [29]. Additional pairing to the middle and the 3' end of gga-miR-150 was also observed, extending from one individual match to seven consecutive matches, for sites s4 and s2, respectively (Fig. 3A). We generated MYB mutant pRL-TK vectors, each containing only one of the four mutated target sites (mt1 to mt4), and one mutant containing all four mutated target sites (mt1234). The various MYB mutant pRL-TK vectors were used to transfect either avian DF1 fibroblasts [64] or human 293FT cells, as described above. Avian DF-1 fibroblasts were used to assess gga-miR-150 site usage in the homologous system. We used human 293FT cells, originating from the HEK-293T cell line, as a reference heterologous system, because the HEK-293T cell line has been used to identify miR-150 target sites in mice, humans and zebrafish [16,37]. We then carried out reporter assays. The same reporter activity patterns were observed in both systems, demonstrating that the observed pattern of target site usage resulted from intrinsic characteristics of chicken c-*myb *3'UTR. We found that pmiR-150 did not repress the activity of the MYB mt1234 reporter or the MYB mt2 construct whereas the activities of MYB mt1, mt3 and mt4 were significantly repressed (by about 40 to 60%) (Fig. 3B). Thus, gga-miR-150 specifically targets the 3'UTR of avian MYB, acting principally through target site s2 in both avian DF-1 cells and human 293FT cells. The use of target site s2 by gga-miR-150 therefore seems to result from the intrinsic properties of the chicken MYB 3'UTR. The observed functionality of avian MYB target site 2 is consistent with previous observations showing that higher levels of 3' base pairing render the site more effective [61,62]. Although both target sites, s2 and s4, display strong base pairing with the gga-miR-150 seed sequence, only site s2, which also displays base pairing for 7 nt at its 3' end, is a functional target for gga-miR-150 (Fig. 3A and 3B). This observation highlights the importance of extended 3' pairing for gga-miR-150.

**Figure 4:**
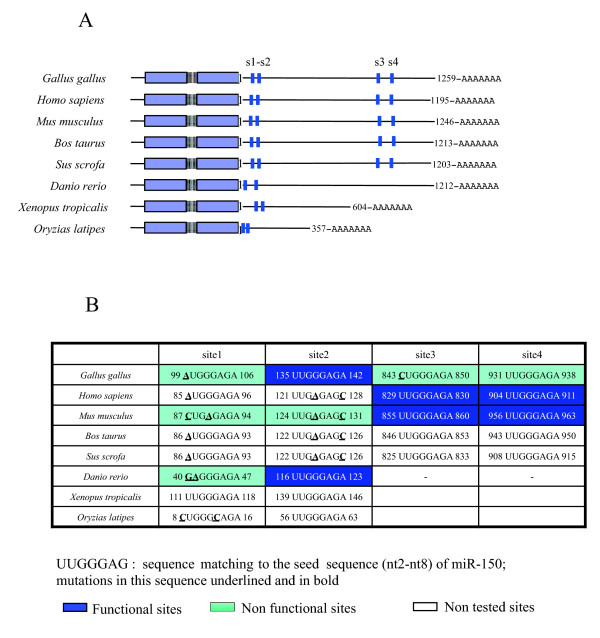
**Putative miR-150 target sites in *MYB *3'UTR conserved through evolution**. The chicken *MYB *3'UTR contains the same four putative miR-150 target sites as observed in mammals, but retains the same functional site as zebrafish. ***(A) ***Schematic representation of *MYB *mRNA through evolution and location of putative miR-150 target sites. ***(B) ***Sequences and function of the different sites through evolution; numbers correspond to the positions of the sites on the respective 3'UTRs (nt1 = first nt downstream from the stop codon). The following sequences were used for this work: *Gallus gallus*, NM_205306.1; *Homo sapiens*, NM_001130172.1; *Mus musculus*, NM_010848.3; *Bos taurus*, NM_175050.1; *Sus scrofa*, XM_001928929.1; *Danio rerio*, BC059803.1; *Xenopus tropicalis*, BC16576.1; *Oryzias latipes*, NM_001104689.1.

Recent studies have demonstrated that MYB targeting by miR-150 has been conserved throughout evolution, as it is observed in mice, humans and zebrafish [16,37]. However, analysis of the targeting of MYB 3' UTR by miR-150 in the various species in which functional target sites have been validated has shown that miR-150 targeting seems to be dependent on the use of different target sites. Synergy between two target sites located in the last third of the 3' UTR of the human and murine *c-myb *genes is required for the regulation of these genes [16,37], whereas only one target site about 120 nt downstream from the *c-myb *stop codon is functional in chicken and zebrafish (Fig. 4). Detailed analysis of the sequence of the 3' UTR of MYB from various species showed that the short 3'UTRs of MYB (frog and medaka) and the 3' UTR of MYB orthologs of zebrafish contained two miR-150 target sites close together in the proximal region, less than 150 nt downstream from the stop codon, and that the large 3'UTR of chickens and mammals contained two additional miR-150 target sites located close together, between 800 and 970 nt from the stop codon (Fig. 4A). However, not all these sites appeared functional and most non-functional ones were mutated, with the exception of site 4 in chicken (Fig. 4B). Site 2, for example, which is functional in chicken and zebrafish and has two conserved mutations in mammals, is not functional in mouse [37] (Fig. 4B). Surprisingly, despite the presence of sequence changes in site 2 rendering it non-functional in mammals (Fig. 4B), the 3' end sequences of this site identified in chicken and zebrafish were found to be strongly conserved throughout evolution, from frogs to humans (Additional file 3), but were not sufficient for compensatory pairing [62]. We identified no functional role for target sites 3 and 4 in chicken MYB, whereas gga-miR-150 targeted and repressed MYB mt3 or mt4 reporter constructs but not MYB mt2 reporter construct. The mutation of target site 2 completely abolished the repressor effect of gga-miR-150, as observed with the mt1234 reporter construct, in which all target sites were mutated. By contrast, miR-150 acts at sites s3 and s4 in mice and humans (Fig. 4B)[16,37]. The mutation introduced into target site 3 of the chicken MYB, resulting in the sequence involved in base pairing to the miR-150 seed sequence being one nucleotide shorter than in mammals (Fig. 4B, Additional file 3), may account for the loss of function of this mutant site. This mutation may also affect the function of conserved site 4, which was non-functional despite a perfect seed match of 8 nt. The observed non-functionality of target site 4 suggests that this site may be functional only in synergy with target site 3, as observed in mammals [16,37] (Fig. 3 and 4).

Features common to all miR-150/MYB target sites have been identified in studies of different species: a perfect seed match of eight to nine nucleotides is required, with either additional 3' pairing or synergy with another closely located site. *c-myb *is the only member of the MYB family of transcription factor genes targeted by miR-150.

Despite the sequence conservation between avian and mammalian MYB 3'UTRs making it possible to align these sequences, our observations suggest that the intrinsic characteristics of the avian MYB 3'UTR result in avian miR-150 selecting a target site different from its mammalian orthologs.

The intermediate evolutionary position of chickens in the vertebrate group may provide insight into the evolution of MYB/miR-150 target sites. Chicken has retained the same functional target site as zebrafish and, probably, frogs. This site appears to be the principal target site in chicken, despite the presence of the putative mammalian target sites in the chicken MYB.

## Conclusion

Our study of the avian MYB/gga-miR-150 interaction shows that, despite the involvement of *c-myb *in development and haematopoiesis in a wide range of organisms, from zebrafish to humans, and its regulation principally through miR-150, the conservation of miR-150 target site functionality observed between chicken and zebrafish does not extend to mammals. An additional set of functional target sites has evolved in mammals, reflecting target site adaptation in these organisms.

## Methods

### Cell lines

We used three chicken cell lines and one human cell line: the DF-1 chicken fibroblast cell line, the Marek's disease virus-induced lymphoma-derived MSB-1 cell line and PA9 chicken T cells, the HEK-293FT (293FT) human cell line. The DF-1 cell line was cultured in DMEM (Lonza France) supplemented with 10% foetal bovine serum and 5% chicken serum (Invitrogen-Life Technologies). The MSB-1 and PA9 cell lines were cultured in RPMI-1640 medium (Lonza France) supplemented with 10% foetal bovine serum and 5% chicken serum. The three cell lines were maintained at 41°C in an atmosphere containing 5% CO_2_. The 293FT cell line (Invitrogen-Life Technologies) is a fast-growing variant of the HEK-293FT that stably expresses SV40 TAg and the neomycin resistance gene from pCMVPORT6AT.neo. 293FT cells were cultured in DMEM supplemented with 10% foetal bovine serum and were maintained at 37°C in an atmosphere containing 5% CO_2_.

### Plasmid construction

All primer sequences are listed in Additional file 2. The avian synthetic premiR-150 DNA sequence was obtained by overlap extension with primers 610 and 611, by polymerase chain reaction (PCR). The resulting PCR product was inserted into the pGEM-T Easy vector (Promega), digested with *Kpn*I and *Xho*I and inserted into the pcDNA3 expression vector downstream from the cytomegalovirus (CMV) promoter (pmiR-150). A mutated premiR-150 DNA with a deletion of one cytosine residue from the seed sequence of miR-150 was also constructed with primers 612 and 611, using the same protocol (pmiR-150mut). The 3' untranslated region (UTR) fragment (1259 nt from the stop codon) of the avian MYB cDNA (GenBank accession number NM_205306.1), which contains four putative miR-150 target sites, was amplified by PCR with primers 682 and 683, using genomic DNA from the thymus of a four-week-old chicken as the template. The PCR product was inserted into the *Not*I site of the *Renilla *luciferase gene 3'UTR region in a pRL-TK vector (Promega), giving the MYB wild-type (WT) reporter. We generated MYB mutant reporters, containing mutations (poly-T or A-replacing sequences) generating mismatches within the "seed region" of miR-150, using a PCR-based protocol, as previously described [65]. Mutations were introduced into target site 1 directly, via the sequence of primer 806, in a one-step PCR amplification also involving primer 683. Mutations of target sites 2, 3 and 4 were introduced by overlap extension, using primer pairs 697/698, 699/700, 701/702 (Additional file 2), respectively, in an initial amplification step, followed by a second step with primers 682 and 683 (Additional file 2). All intermediate and final constructs were checked by sequencing with appropriate primers.

### Isolation of lymphoid cells ectopically producing miR-150 or miR-150mut

The stable expression of constructs encoding miR-150 or miR-150mut in PA9 cells was obtained by electroporation with the pmiR-150 and pmiR-150mut plasmids, respectively, using the Amaxa nucleofector device (Lonza): plasmid (2.5 μg) was added to 2 × 10^6 ^cells, with a pcDNA control used in parallel, and cells were cultured in complete RPMI-1640 medium without selection for 24 hours. Cells were then selected in complete RPMI-1640 medium supplemented with 1 mg/ml G418 for three weeks.

### Luciferase assay

For the luciferase assay, pcDNAMLuc, carrying the firefly luciferase gene under the control of the CMV promoter, was used for cotransfection, to control for transfection efficiency.

PA-9 and MSB-1 cells were electroporated with an Equibio "EasyjecT Plus" electroporator (single pulse, 400 V, 1500 μF) and aluminium electrodes (4-mm cuvette, Eurogentec). For all assays, 5 × 10^6 ^cells were electroporated in the presence of 40 μg of pRL-TK_MYBWT and 500 ng of pcDNAMLuc in serum-free RPMI 1640 medium. After electroporation, cells were plated in 6-well plates, each well containing 2.5 ml of RPMI-1640 medium supplemented with serum, and cultured for 24 hours at 41°C.

DF-1 and 293FT cells were seeded in 96-well plates (2.5 × 10^4 ^cells per well). They were cultured for 24 hours before cotransfection with reporter plasmids (100 ng of pRL-TK_MYBWT or mutated and 1.25 ng of pcDNAMLuc) and 300 ng of effector plasmids (pmiR-150 or pmiR-150mut or pcDNA control), using Lipofectamine 2000 (Invitrogen-Life technologies) according to the manufacturer's protocol.

Luciferase assays were performed 24 hours after transfection, with the Dual-Luciferase Reporter assay system (Promega). *Renilla *luciferase activity was normalised with respect to firefly luciferase activity. Each transfection reaction was repeated in triplicate for each set of conditions and the experiment was carried out at least three times. Mean relative luciferase activity is presented. The significance of differences between effector and reporter constructs was assessed with Student's t-test. We considered *P *values < 0.05 to be statistically significant.

### Northern blotting

Total RNA was extracted from 5 × 10^6 ^cells with Trizol reagent (Invitrogen-Life Technologies), according to the manufacturer's protocol. Briefly, 15 μg of RNA was subjected to electrophoresis in a 15% acrylamide gel and transferred onto a nylon membrane (Macherey-Nagel Porablot Nylon Activated). The membrane was crosslinked by exposure to UV light (Stratalinker, Stratagene). The membrane was prehybridised for 1 h and then hybridised overnight in Perfect Hyb TM Plus hybridisation buffer (Sigma), at 50°C, with a 20 nM ^32^P-5' end-labelled DNA oligonucleotide probe complementary to the miRNA, and then washed in low-stringency wash buffer. Blots were analysed by phosphorimaging with a Storm 840 (Amersham).

### Sequence analyses and target prediction

The online target prediction algorithm Targetscan [66]http://www.targetscan.org was used to list potential miR-150 target sites from mammalian, frog and chicken MYB 3'UTRs. Additional Blast analysis of the miR-150 sequence against the MYB 3'UTR sequence of each species analysed was carried out with the accessory application "local blast" available in BioEdit version 7.0.5 sequence alignment software.

## Authors' contributions

DR conceived and designed the study. DR and GD co-ordinated the study. AGM and GD contributed to the design of the experiments. AGM, NR and DC carried out the experiments. BM screened the spleen and blood leukocyte small-RNA libraries, isolated gga-miR-150 and helped with northern-blot analysis. AGM contributed to the interpretation of the data and to the drafting of the manuscript. GD and DR wrote the manuscript. All authors read and approved the final manuscript.

## Supplementary Material

Additional file 1 **Hairpin structures of threedifferent mammalian pre-miR-150 sequences and of the synthetic *Gallus gallus *pre-miR-150**. The mature miR-150 is shown in italics and the seed sequence is shown in red. Click here for file

Additional file 2 **Table of primer sequences**. Click here for file

Additional file 3 **Predicted binding structures for miR-150 and the four putative target sites (s1, s2, s3, s4) from different species, through evolution**. The seed sequence (nt2 to nt8) of miR-150 is shown in red, bold. The seed sequence nucleotides involved in pairing are underlined; nucleotides involved in pairing outside the seed sequence are shown in blue, bold. The target sites nucleotides involved in pairing are shown in bold. Click here for file
