# Supplementary material for: The chicken miR-150 targets the avian orthologue of the functional zebrafish MYB 3'UTR target site
Source: BMC Mol Biol. 2010 Sep 2;11:67. doi: 10.1186/1471-2199-11-67 (PMC2940766; doi:10.1186/1471-2199-11-67)
Supplement: Additional file 1 — Hairpin structures of threedifferent mammalian pre-miR-150 sequences and of the synthetic Gallus gallus pre-miR-150. The mature miR-150 is shown in italics and the seed sequence is shown in red. [file 1471-2199-11-67-S1.PDF]

```
5'c      u  -             ac   u        u -   g
   ucccca gg cccugucuccca  ccu guaccag g cug g
   |||||| || ||||||||||||  ||| ||||||| | |||
   aggggu cc gggacaggggu   gga caugguc c gac c
3'c      -  a             cc   -        c a   u
```

Homo sapiens

```
5'c        g             ac   u        u -   u
   uucucaag cccugucuccca  ccu guaccag g cug g
   |||||||| ||||||||||||  ||| ||||||| | |||   c
   agggguuc gggacaggggu   gga caugguc c gac c
3'c        a             cc   -        c a   u
```

Rattus norvegicus

```
5' cuc     u  c  -             aacc           u  g
       ucucc ca gg cccugucuccc    cuuguaccag gu ugu
       ||||| || || |||||||||||    |||||||||| || || c
       agggg gu cc gggacggaggg    ggacaugguc ca acu
3' ccu     -  -  a             gcau           c  g
```

Bos taurus

```
5'cuc      u  -             acc          u -   g
   uccuca gg cccugucuccca   ccuguaccag g cug u
   |||||| || ||||||||||||   |||||||||| | |||
   aggggu cc gggacaggggu    ggacaugguc c gac c
3'--c      -  a            -cc           c a   u
```

Synthetic *Gallus gallus*
