# Supplementary material for: The chicken miR-150 targets the avian orthologue of the functional zebrafish MYB 3'UTR target site
Source: BMC Mol Biol. 2010 Sep 2;11:67. doi: 10.1186/1471-2199-11-67 (PMC2940766; doi:10.1186/1471-2199-11-67)
Supplement: Additional file 2 — Table of primer sequences. [file 1471-2199-11-67-S2.PDF]

Primer sequences

Primers	Sequence 5'==> 3'
**miR-150**	
610 miR 150 f	CAGCTCGAGCTCTCCTCACGGCCCTGTCTCCCAACCCCTGTAC**CAGTGCTGGTCTCAGA**
611 miR-150 r	CAGGGTACCGTCCCCAGGTCCCTGTCCCCCAGGCCTGTACCAGGG**TCTGAGACCAGCACTG**
612 miR-150 mut f	CAGCTCGAGCTCTCCTCACGGCCCTGTCTCC-AACCCCTGTAC**CAGTGCTGGTCTCAGA**
**c-Myb 3'UTR**	
682 f	CAGGCGGCCGCGACTTCTCCGGAGAAG
683 r	CAGGCGGCCGCAATTAAGTGCAGGTGAAGC
**c-Myb 3'UTR m1**	
806 f	CAGGCGGCCGCCGGAGAAGCATTATGGTTGGCAAACACTCCACGTTCGCTGGGAAGTCCCTG TTCCTCTAAACAAGGACTTTTTGTGAAtttttttCGAGCCTATCTTTG
**c-Myb 3'UTR m2**	
697 r	**AATGCACTTCGTGC**ttttttttCTGTTGTACCAC
698 f	**GCACGAAGTGCATT**TAGTCACTGAGC
**c-Myb 3'UTR m3**	
699 r	**CACACATTGAGAC**AATTAAAACAAAAAAAAAAtttttttGAACAGTGTTC
700 f	**GTCTCAATGTGTG**GTTTTGTTTCACACTGG
**c-Myb 3'UTR m4**	
701 r	**CGAGACAGTG**ttttttttAACATATGTAGG
702 f	**CACTGTCTCG**TTCGTGTTGTCTGTTTTGTTCC

f: forward, r: reverse; restriction sites underlined; overlapping nucleotides in bold; mutated nucleotides in lower case
