# Supplementary material for: The chicken miR-150 targets the avian orthologue of the functional zebrafish MYB 3'UTR target site
Source: BMC Mol Biol. 2010 Sep 2;11:67. doi: 10.1186/1471-2199-11-67 (PMC2940766; doi:10.1186/1471-2199-11-67)
Supplement: Additional file 3 — Predicted binding structures for miR-150 and the four putative target sites (s1, s2, s3, s4) from different species, through evolution. The seed sequence (nt2 to nt8) of miR-150 is shown in red, bold. The seed sequence nucleotides involved in pairing are underlined; nucleotides involved in pairing outside the seed sequence are shown in blue, bold. The target sites nucleotides involved in pairing are shown in bold. [file 1471-2199-11-67-S3.PDF]

```
S1                                                  S2

CAAGGAGUUUUUGUGAAUGGGAGACGAGCCUAUCUUUGUUGUGGUACAACAGUUGGGAGAGCA        Gallus gallus
3'GUGACCAUGUCCCCAACCCUCU 5'              3'GUGACCAUGUCCCCAACCCUCU 5'

AUGAAACUUUUCAUGAAUGGGAGAAGAACCUAUUUUUGUUGUGGUACAACAGUUGAGAGCAGC        Homo sapiens
3'GUGACCAUGUUCCCAACCCUCU 5'              3'GUGACCAUGUUCCCAACCCUCU 5'

GUGGAGCGCUCCAUGGCUGAGAGAAGAGCCUGAUUUUGUUGUGGUACAACAGUUGAGAGCAGC        Mus musculus
3'GUGACCAUGUUCCCAACCCUCU 5'              3'GUGACCAUGUUCCCAACCCUCU 5'

 AUGAGACUUUUCAUGAAUGGGAGAAGAGCCUAUUUUUGUUGUGGUACAACAGUUGAGAGCAGC       Bos taurus
3'UGUGACCAUGUUCCCAACCCUCU 5'             3'UGUGACCAUGUUCCCAACCCUCU 5'

GUGAAACUUUUCAUGAAUGGGAGAAGAGCCUAUUUUUGUUGUGGUACAACAGUUGAGAGCAGC        Sus scrofa
3'GUGACCAUGUUCCCAACCCUCU 5'              3'GUGACCAUGUUCCCAACCCUCU 5'

AUUCUGAAAAGAAGUAAAAAGAGGAACAGUCCCUACACUCGUGGUACAUUAUUUGGGAGAGGC        Danio rerio
                                          3'GUGACCAUGUUCCUAACCCUCU 5'

AACGCUGGGCAGACACAGCAUCACAGGUCUCUGUGUUUUUGUGGUACAACAGUUGGGAGAGGC    Oryzias latipes
                                          3'GUGACCAUGUUCCCAACCCUCU 5'

AAACGUUGGGAAAUUUGUUCAUGAUUGGGAGAAAUUUGUUGUGGUACAACAGUUGGGAGAAGC Xenopus tropicalis
        3'GAGACCAUGUUCCCAACCCUCU 5' 3'GAGACCAUGUUCCCAACCCUCU 5'


S3                                                  S4

UUUUUGAACACUGUUCCUGGGAGAUUUUUUU----ACAGCAUGUAUUGCACCUUCAUAUGUUUGGGAGAACA  Gallus gallus
3'GUGACCAUGUCCCCAACCCUCU 5'                 3'GUGACCAUGUCCCCAACCCUCU 5'

ACAUUUGAAAACUUGUUUGGGAGACUCUGCA----UUAUACAAGCAUGCGUUGCACUUCUUUUUUGGGAGAUGU Homo sapiens
3'GUGACCAUGUUCCCAACCCUCU 5'                   3'GUGACCAUGUUCCCAACCCUCU 5'

UAAUUGGAAAACUUAUUUGGGAGAAUUUUGC----UAAAAGCAUUGCACUUCUUUUUCUUUUUUUGGGAGAUUU Mus musculus
3'GUGACCAUGUUCCCAACCCUCU 5'                   3'GUGACCAUGUUCCCAACCCUCU 5'

ACAUUGGAAAACUUAUUUGGGAGACUUUGCA----UUAUAAGAGGAUGCAUUGCACUUCUUUAUUGGGAGAUCU   Bos taurus
3'UGUGACCAUGUUCCCAACCCUCU 5'                 3'UGUGACCAUGUUCCCAACCCUCU 5'

ACAUUGGAAAACUUGUUUGGGAGAUUUUGCA----UUAUAAGAGCAUGCAUUGCACUUCUUUUUUGGGAGAUCU   Sus scrofa
3'GUGACCAUGUUCCCAACCCUCU 5'                   3'GUGACCAUGUUCCCAACCCUCU 5'
```
